# The Royal Sanitary Institute

**Published:** 1905-05-20

**Authors:** 


### THE ROYAL SANITARY INSTITUTE.

The Royal Sanitary Institute of Great Britain has just put forth an appeal for the modest sum of £15,000 as a means of removing its operations to more commodious premises than those of its present habitation in Margaret Street, and of extending its work in the many directions in which such extension is called for. The Institute has an interesting history, and one very typical of the vitality of private and individual work in this country, as well as of the magnitude which may come into existence as the consequence of a very small beginning. It was founded in the early seventies by the late Dr. Lory Marsh, a Nottingham physician, who had migrated to London, and who thought it would be useful to establish a centre of examination for persons desirous to engage in sanitary work as surveyors and inspectors. Among those who were active in the first stage of its proceedings, and most of whom have now been taken away from us, may be mentioned General H. Y. D. Scott, the architect of the Royal Albert Hall, and the patentee of one of the many methods of dealing with sewage which raised vain hopes in the minds of their promoters, Sir Benjamin Ward Richardson, Mr. Rogers Feld, Mr. Eassie (the first honorary secretary of the Cremation Society) and a few others. The new Society speedily instituted examinations, and these were soon resorted to by a considerable number of candidates, but the fees were necessarily very small, and the maintenance of a high standard as measured by the sanitary knowledge of the day had very little influence in the direction of obtaining funds. Things came to such a pass that a few of the founders or original members were at last compelled to contribute £10 each to keep the doors open, and they did so as the pecuniary qualification for life membership. A crisis in the affairs of the young society having been thus averted, fortune began slowly to smile upon its efforts, and it was ultimately able to coalesce with the museum which had been founded in memory of the late Dr. Parkes, and materially to enlarge the sphere of its operations, obtaining, by this alliance, the active and energetic help of the late Dr. Vivian Poore. The late Duke of Cambridge accepted the presidency, and took a warm interest in the work, and on his decease he was succeeded by the Duke of Northumberland, who now fills the office, under the patronage of his Majesty the King, who has lately graciously signified his pleasure that the Institute should add the prefix of "Royal" to its former designation. The Parkes Museum, which, besides being a most important adjunct to the teaching power of the Institute, is open to the public without charge, contains a collection of models and apparatus designed and arranged for instruction in the principles and practice of hygiene and sanitary science, and is used for this purpose, not only by the Institute itself, but by many colleges and schools training candidates for public health offices, by technical schools, by secondary schools, and by several other public bodies. The Institute has also collected and arranged a large and valuable library, has instituted courses of training for sanitary officers, meat inspectors, school teachers, and others; and also examinations, which have been approved and adopted for certain purposes by the War Office, the Local Government Boards of England, Scotland, and Ireland, and by the London Education Committee. It has co-operated with various municipal and educational authorities in the Colonies in instituting sanitary lectures and training, and has established, under its control, and with the sanction and assistance of the Colonial Office, examinations in the principal colonial centres. In England it holds a migratory annual meeting and museum at some great centre of population, besides smaller provincial meetings at other times, and it publishes a very excellent journal, which will for the future be issued monthly, and in which all important contributions to the meetings, with reports of the discussions following them, are contained. In all these directions the good work accomplished in the past is likely to be increased in the future, and the Council has now an opportunity of renting a site in Westminster admirably adapted for its requirements and its development. The educational work so well carried out in Margaret Street has been of imperial as well as of national importance, and has done so much to promote the improvement of the public health, and to provide facilities for the higher studies of sanitary science, that the Council have abundant justification for coming to the public for assistance. Their claims on the score of past and prospective usefulness are enhanced by the fact that their management hitherto has been economical and prudent, and that they are able to meet a large portion of the anticipated expenditure from funds which have been husbanded for the purpose.

